# RNA-seq and ChIP-seq as Complementary Approaches for Comprehension of Plant Transcriptional Regulatory Mechanism

**DOI:** 10.3390/ijms21010167

**Published:** 2019-12-25

**Authors:** Isiaka Ibrahim Muhammad, Sze Ling Kong, Siti Nor Akmar Abdullah, Umaiyal Munusamy

**Affiliations:** 1 Laboratory of Plantation Science and Technology, Institute of Plantation Studies, Universiti Putra Malaysia, Selangor 43400, Malaysia; muhammadii108@gmail.com (I.I.M.); szeling0923@gmail.com (S.L.K.); yalyagu@gmail.com (U.M.); 2 Department of Agriculture Technology, Faculty of Agriculture, Universiti Putra Malaysia, Selangor 43400, Malaysia

**Keywords:** RNA-sequencing, ChIP-sequencing, transcriptome, transcriptional regulatory mechanism, data integration

## Abstract

The availability of data produced from various sequencing platforms offer the possibility to answer complex questions in plant research. However, drawbacks can arise when there are gaps in the information generated, and complementary platforms are essential to obtain more comprehensive data sets relating to specific biological process, such as responses to environmental perturbations in plant systems. The investigation of transcriptional regulation raises different challenges, particularly in associating differentially expressed transcription factors with their downstream responsive genes. In this paper, we discuss the integration of transcriptional factor studies through RNA sequencing (RNA-seq) and Chromatin Immunoprecipitation sequencing (ChIP-seq). We show how the data from ChIP-seq can strengthen information generated from RNA-seq in elucidating gene regulatory mechanisms. In particular, we discuss how integration of ChIP-seq and RNA-seq data can help to unravel transcriptional regulatory networks. This review discusses recent advances in methods for studying transcriptional regulation using these two methods. It also provides guidelines for making choices in selecting specific protocols in RNA-seq pipelines for genome-wide analysis to achieve more detailed characterization of specific transcription regulatory pathways via ChIP-seq.

## 1. Introduction

The transcriptome defines the functional element in a genome as it encompasses the complete set of coding and non-coding RNA molecules present in a single cell or a population of cells [1]. The ultimate expression of a subset of genes into complementary RNA transcripts would designate a cell’s identity and the control of the biological activities within the cell [2]. Transcriptome profiling therefore can greatly facilitate the understanding of a functional genome via characterization of the gene structures, identification of the alternative splicing events, as well as detection of the dynamic regulation of transcripts in various tissues during development, diseased, or stressed conditions [3].

Ever since they were first introduced in 2005, high throughput next-generation DNA sequencing (NGS) technologies have revolutionized the transcriptomics field through massively parallel sequencing of complementary DNA (cDNAs) derived from a transcript population. This important application of NGS termed RNA-sequencing (RNA-seq) [4,5] has overcome several limitations posed by generally used microarray technologies, including not requiring prior knowledge of the genome or sequence of interest, which enables genome-wide unbiased detection of both known and novel transcripts [3]. Single nucleotide-resolution RNA-seq data can also enhance the detection of alternative splicing events and isoform expression. Reanalyzing RNA-seq data in relation to any new genome or datasets that become available in future also can be easily achieved [6]. On the other hand, microarrays inherently exhibit cross-hybridization results in high background noise and have a limited dynamic range of detection, for example in identification of low-abundance transcripts [7]. Due to its distinct advantages and rapid decrease in the per-base costs, together with the application of multiplexing strategies, RNA-seq methods have mostly displaced hybridization-based methods as the preferred option for gene expression studies [6]. With constantly improving RNA-seq techniques and platforms for bioinformatics analysis, RNA-seq has been widely adopted in the analysis of both prokaryotic and eukaryotic transcriptomes as in the studies of bacterial pathogens [8,9,10], livestock [11,12,13,14], and human cancer and disease [15,16,17,18].

Since the initiation of the oneKP project, which aims to sequence 1000 of plant transcriptomes, RNA-seq has been extensively applied to transcriptome studies of a wide range of economically important crop plants [19,20,21,22]. Moreover, integration of RNA-seq with different molecular biology and biochemical techniques has allowed deeper exploration of various aspects of the transcriptome in plants, such as miRNA-seq [23], Ribo-seq [24], HITS-CLIP/CLIP-seq [25], and GRO-seq [26].

Protein–DNA binding interactions play key roles in gene regulatory and expression processes such as replication, splicing, transcription, and DNA repair. To predict the accuracy of modified histones and bound proteins, functional assays were developed, such as electrophoretic shift mobility assays (EMSA) [27,28], DNA microarrays [29], yeast one-hybrid studies [30,31], and chromatin immunoprecipitation, followed by microarray, also termed ChIP-chip [32]. Chromatin immunoprecipitation, followed by sequencing (ChIP-seq) assays, have become an indispensable next generation technique for detecting in vivo interactions of DNA target sites against their corresponding transcription factors (TFs), epigenetic histone modifications, as well as chromatin remodeling. Chromosome structure and function is largely determined by nucleic acids interactions with specific proteins [33]. ChIP-seq is, so far, the best technique to study these interactions because of its improved signal-to noise ratio and genomic sequence information [34]. ChIP-seq nomenclature has been reported in different forms to suit different investigators’ research goals. For instance, ChIP quantitative polymerase chain reaction (ChIP-qPCR) was developed to be a robust method to analyze ChIP-data via different normalization strategies [35]. In contrast, Nano-ChIP-seq has been used to study protein DNA interactions where little source of DNA is available [36], which is necessary because ChIP-seq was originally proposed to use a large number of cells.

The ‘big data’ generated by many high-throughput technologies often tend to be noisy and contains various sources of unwanted variance and procedural artifacts. It is a challenge for the accurate analysis of extraordinary data volumes to identify true signals, combine variable data types, and understand their relationships [37]. When designing integrated Omics (ChIP-seq and RNA-seq) experiments, RNA-seq can be performed prior to ChIP-seq. In this way, the most enriched TF in differentially expressed genes (DEGs) revealed by RNA-seq, such as in studies involving biotic or abiotic stress treatments, are considered as targets for ChIP-seq assay either by raising custom antibodies against the TFs [38] or through transgenic expression against the tagged TFs (tags like FLAG, green fluorescent protein (GFP), and Glutathione S-transferase (GST), etc.) in model plants [39]. Moreover, independent ChIP-seq can be carried out based on TFs that have substantial literature information and subsequently comparing with an independent RNA-seq assay under the same biological treatment on the same plant. Combining ChIP-seq and RNA-seq assays can show agreement between both findings, revealing more information about a TF by either discovering a new function or a new set of genes for the same function [40]. Assays of transposase accessible chromatin [41] (ATAC-seq) can also measure how much chromatin can be accessed for peak enrichment from ChIP-seq assay [42] and therefore, it can be accompanied with ChIP-seq assay.

In this review, we will discuss how ChIP-seq can strengthen information generated from RNA-seq in elucidating the role of transcription factors. To be precise, we discuss how a combination of ChIP-seq and RNA-seq data can help to unravel the transcriptional regulatory network. RNA-seq essentially serves as the gene discovery tool for identifying specific transcription factors based on their expression profiles and the profile of potential target genes. The ChIP-seq is potentially useful to validate transcription factor target (downstream) genes interaction with potential link with certain physiological or biochemical processes. This review also discusses the transformation of RNA-seq and ChIP-seq assays over time, together with a review of the basic steps required for plant system, highlighting the most recent applications in different plant species. We also review the basic characteristics of RNA-seq and ChIP-seq data analysis pipelines. We then provide examples of genome-wide identification of transcription factor co-regulated genes by RNA-seq and ChIP-seq, which highlight the potential of such studies in elucidating transcriptional regulatory network in important biological processes in plants. These examples will show how combining these tools will help in addressing hormonal response like jasmonic acid in Arabidopsis [1], gibberellic acid in rice [2], and the developmental stage effect in maize [3] to reveal some important insights on their transcriptional regulatory mechanisms. We also introduce the third-generation sequencing, which expands the application of sequencing technology due to the longer read length offering higher capability in sequence assembly and identifying sequence variance in RNA-seq.

## 2. RNA-seq Platform Selections

**Figure 1:**
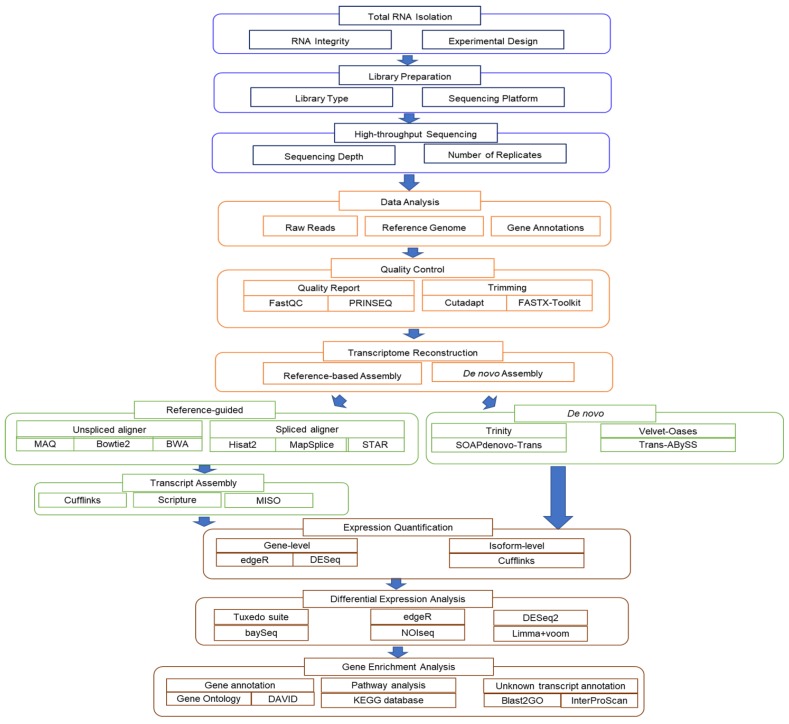
General RNA-seq analysis pipeline. The workflow typically starts with total RNA extraction depending on experimental design and RNA integrity. The library preparation step relies on the selection of sequencing platform and library type, while sequencing depth and number of replicates can impact the downstream sequencing output analysis processes. RNA-seq data analysis generally requires inputs such as raw sequencing reads, reference genome sequences, and gene annotations. Next is examination of raw data quality and perform poor read trimming, transcriptome assembly, and expression quantification. Finally, differential expressed genes (DEGs) must be identified and interpreted through gene enrichment analysis. Each step in the data analysis has several representative tools, as highlighted.

There are several commercially available deep sequencing platforms for RNA-seq, such as Ion Torrent, PacBio, and Illumina [43]. Currently, the HiSeq series of sequencers from Illumina is the most widely deployed sequencing platform due to its ability to produce a high data output with low sequencing errors. In view of the variation in data quality and quantity achieved from different deep sequencing platforms and the downstream interpretation processes, the selection of a suitable sequencing platform based on the research goals is an initial key step before starting an RNA-seq experiment. For instance, Illumina Hiseq can produce short reads (50–250 bp), while PacBio generates longer reads (4200–8500 bp). Longer reads will ease the de novo transcriptome assembly process and the detection of alternative splice isoforms compared with short reads. Additionally, paired-end reads (sequencing from both ends of a fragment) are attainable with Illumina instruments but not with Ion Torrent [2,43,44]. Paired-end reads uncover sequence from both ends of the cDNA fragment and accelerate the inspection of splicing variants, chimeric transcripts, and indels [45]. Figure 1 shows the summarized RNA-seq workflow comprising of the wet laboratory works (RNA extraction, library preparation, and sequencing) and the dry laboratory works (in silico RNA-seq data analysis). We will go through each of these steps in more detail in the text below.

In general, RNA-seq experiments start with total RNA isolation and selection of a specific RNA population, such as messenger RNA (mRNA) or microRNA (miRNA), before subjecting samples to a fragmentation step. Next, the short RNAs are converted to a cDNA library and each cDNA fragment is ligated with platform-specific adaptors at one or both ends in order to capture the fragments on a solid support. Millions of reads from one end (single-end sequencing) or both ends (pair-end sequencing) are retrieved by parallel sequencing of millions of cDNA fragments in different NGS platform. Read lengths can vary depending on the sequencing chemistry and technology. The resulting reads will either align to a reference genome or transcriptome or de novo assembled to produce a genome-wide transcription landscape [6,46]. The comprehension of the generated data to resolve the primary research questions characterize the success of an RNA-seq study. Thus, it is crucial to consider upfront several key points before conducting an RNA-seq experiment, such as the selection of the library type, the number of biological and technical replicates, and the depth of sequencing across the transcriptome [47].

Determination of the number of biological and technical replicates required in an RNA-seq experiment varies with the technical biases and the heterogeneity of each experimental system. While reproducibility of RNA-seq data across lanes and flow cells is generally high, biological replication is mandatory for population inferential analysis [48]. Optimal data interpretation can be achieved by reducing the data variability with duplicate or triplicate experimental datasets [49]. The optimal sequencing depth, which means the number of sequencing reads for a given sample, is strongly governed by the aims of the study. Generally, the required sequencing coverage depends on several factors, including reference genome size, gene expression level, and specific application of interest using the data generated. In this review, we will provide an overview of a typical RNA-seq experiment and steps involved in the bioinformatics analysis.

## 3. RNA-seq Workflow (Wet Laboratory)

### 3.1. Total RNA Isolation

High quality RNA is a prerequisite for a successful RNA-seq experiment. RNA integrity number (RIN), a measuring unit produced by Agilent Bioanalyzer, is an unofficial standard used to estimate the integrity of RNA before proceeding with library preparation step. RIN ranges from 1 to 10, with 10 being the highest score for samples with minimal degradation. RIN < 6 indicates low quality RNA that can introduce substantial biases into the final sequencing results [2]. But for plant materials, a good RIN number can be lower depending on species and tissue types. For fluorometric quantification of the RNA input, Thermo Fisher Scientific Qubit or Nanodrop is the most commonly used fluorometer [50].

### 3.2. Library Preparation

The second step in RNA-seq is the construction of an RNA-seq library. It starts with enrichment or depletion of the total RNA pool for that desired RNA species. In most cell types, RNA can be divided into different populations comprised of ribosomal RNA (rRNA), transfer RNA (tRNA), non-coding RNA (ncRNA), and messenger RNA (mRNA), which is the common interest in most transcriptome studies. Deep sequencing without removal of rRNAs that occupy 80% of the RNA population will reduce the depth of sequence coverage and limit the detection of lowly expressed transcripts [47]. The common practice before library construction is to enrich the mRNA by selecting the poly(A) RNAs. Poly(A) RNA can be isolated using magnetic or cellulose beads coated with poly-T oligos. Alternatively, rRNA depletion can be carried out through duplex-specific nucleases treatment or commercial kits such as Ribo-Zero (Illumina), NEBNext^®^ (New England BioLabs) or RiboMinus (Thermo Fisher). The technical limitations and biases of each approach need to be discerned in order to choose the most appropriate method for library preparation. For example, if one aimed at the exploration of noncoding RNA including pre-mRNA, then ribo-depletion libraries are a more appropriate choice than poly(A) libraries. In recent years, small RNA which includes microRNA (miRNA), small interfering RNA (siRNA), and piwi-interacting RNA (piRNA) have gained great interest among plant researchers to profile and characterize their functions in post-transcriptional regulation. Therefore, several commercially available isolation kits have been developed to capture these short and lowly abundant transcripts based on size fractionation method through gel electrophoresis or silica spin columns [2].

Following poly(A) RNA selection or rRNA removal, RNA molecules need to be fragmented into appropriate sizes (120–200 bp) by enzymatic digestion or chemical hydrolysis under an elevated temperature. In the case of small RNAs, no fragmentation step is needed, and one can directly proceed with adaptor ligation. Once the RNA is cleaved, samples are reverse transcribed into first strand complementary DNA (cDNA) using random primers. After synthesis, the second strand cDNA using DNA polymerase I and RNase H, a single ‘A’ base is added to the end of each cDNA fragments before ligating with the sequencing adapters. The cDNA pool is then purified and amplified to form the final sequencing-ready cDNA library [47]. By having the sequencing adapters ligated to both ends of the cDNA, researchers may perform paired-end sequencing, which sequences the cDNA from both directions (forward and reverse) to produce more reliable sequencing data compared to single-end sequencing.

In the standard library construction protocol described above, the information about the strand orientation of each transcript is lost, which could complicate the identification of overlapping genes transcribed from the opposite strand and particularly in de novo transcript discovery. Subsequently, this could mislead the quantification of global expression of both sense and antisense RNAs [51]. The preferred approach to retain the strand origin is by incorporating deoxy-UTPs (dUTPs) instead of dTTPs during the second strand cDNA synthesis step, which can be selectively digested using uracil-*N*-glycosylase (UDG). Eventually, the remaining first strand cDNA is amplified to yield a strand-specific cDNA library. Zhao et al. (2015) [52] demonstrated that stranded RNA-seq could provide better resolution in estimating the relative abundance of overlapping transcripts expression as compared with the conventional non-stranded RNA-seq.

Through the implementation of barcoding strategy, one can carry out multiplexing of several samples in an analysis which could significantly reduce the per sample cost for large scale projects. Van Nieuwerburgh et al. (2011) [53] compared three different barcoding methods, including pre-PCR, TruSeq, and PALM. For pre-PCR method, the barcode is associated with the 5′ RNA adapter and ligated to the RNA template before performing RT-PCR, while the barcode is incorporated into one of the RT-PCR primers during the library amplification step in TruSeq method. On the contrary, PALM barcoding method ligated the T-tailed barcode adapter to the A-tailed RT-PCR products after the library amplification step to produce a library that is free of barcode-induced PCR bias.

## 4. RNA-seq Workflow (Data Analysis)

After completing the sequencing, the next challenge is dealing with millions of reads generated from each experiment. The conventional analysis pipeline of the RNA-seq data starts with quality checks and preprocessing of the raw sequencing short reads, followed by mapping of the filtered reads to a reference genome sequence or de novo assembly using different de novo transcriptome assemblers. Gene expression levels of all mapped transcripts are then quantified and normalized to define the differential expressed genes. Further analysis of the listed genes, such as alternative splicing analysis, functional annotation, and pathway enrichment analysis, can be carried out using a range of bioinformatic programs. Specialized data analysis workflows can be designed according to individual experimental setups and research aims. In this section, we will briefly discuss the routine RNA-seq data analysis pipeline and related bioinformatics tools in each step.

### 4.1. Quality Control

The preliminary sequencing output is supplied in FASTQ format and is generally contaminated with sequencing artefacts and errors which may arise in library preparation, sequencing, or imaging steps that can ultimately lead to misinterpretation and erroneous conclusions. Therefore, pre-processing and quality control of the raw reads data is mandatory to improve downstream assembly quality and computational efficiency [54]. A Phred quality score (Q score) was assigned to estimate the base call accuracy of the sequencing output. Q30 corresponds to an incorrect base call of 1 in 1000 (99.9%) and serves as the gold standard for quality in read data. Publicly available tools such as FastQC (https://www.bioinformatics.babraham.ac.uk/projects/fastqc/) and PRINSEQ (http://prinseq.sourceforge.net/faq.html) can be used to generate summary statistical reports for sequencing outputs including GC content percentage, base quality and content, level of duplication, sequence quality scores, presence of ambiguous bases, etc. Based on the quality report, further removal or trimming of poor-quality reads, adapter sequences, or demultiplexing can be performed using software tools likes Cutadapt (https://cutadapt.readthedocs.org/en/stable) and FASTX-Toolkit (http://hannonlab.cshl.edu/fastx_toolkit) [1,3].

### 4.2. Transcriptome Reconstruction

The process of identifying all of the transcripts and isoforms that are expressed in a specimen through assembly filtered short reads or read alignments into transcription units is defined as transcriptome reconstruction. Transcriptome assembly can be done using two different strategies: reference-based assembly or de novo assembly. The reference-based approach involves mapping the filtered sequencing reads to an annotated genome or transcriptome followed by transcript assembly. This is relatively less computationally intensive compared with de novo assembly. However, the de novo assembly approach is particularly beneficial when a reference genome or transcriptome is not available. The analysis starts with assembling the sequencing reads into contigs, which will be used as a novel reference transcriptome to align with the raw reads again [3].

### 4.3. Reference-Guided Assembly

Once aberrant reads are eliminated, the RNA-seq data is ready for alignment, with the condition that a reference genome or transcriptome is available. The national centre of biotechnology information (NCBI; https://www.ncbi.nlm.nih.gov/genome/), Ensembl (www.ensembl.org/index.html), and UCSC genome browser (https://genome.ucsc.edu/) are the three most well-known publicly available resources for retrieving the reference genomes and annotation files for a variety of species. There are two major categories of computational programs that have been developed to map the millions of short query sequences to a reference genome or transcriptome precisely with appropriate parameter values at each step of the analysis. The first group is referred as unspliced aligners which includes MAQ [55], Bowtie2 [56], and Burrow Wheelers alignment (BWA) [57], which is a better option for prokaryotic RNA-seq analysis, and in reference, transcriptome mapping as splicing event detection is unnecessary. In contrast, spliced aligners such as HiSat2 [58], MapSplice [59], and STAR [60] are extensively applied in mapping query sequences to the reference genome of eukaryotes. This group of aligners possesses the ability to identify the exon boundaries and align the query sequences that span across introns, which consequently increases the possibility for alternative splicing detection. Despite understanding the intrinsic alignment algorithms, computational infrastructure requirements for each mapping tools to complete the tasks should also be taken into consideration. Typically, the aligned read data is presented in SAM file format and then compressed into binary of SAM (BAM) file format. The alignment file can be viewed and manipulated using SAMtools (samtools.sourceforge.net/) and Picard (https://broadinstitute.github.io/picard/). Integrative genomic viewer (IGV) (http://software.broadinstitute.org/software/igv/) is a high-performance viewer that supports diverse file formats, e.g., SAM, BAM, and Goby. In addition to the ability to display varying level of alignment details depending on the resolution scale, IGV is also able to simultaneously display multiple genomic regions in an adjacent panel [61]. Towards the completion of the alignment step, one can assess the quality of the mapping result using tools like Qualimap 2 (http://qualimap.bioinfo.cipf.es/) and RSeQC (http://rseqc.sourceforge.net/) considering several metrics including percentage of mapped reads, error distributions, and 3′–5′ coverage ratio [2,3,47]. Subsequently, the overlapping reads can be assembled into full length transcripts using RNA-seq analysis packages such as Cufflinks [62], Scripture [63], and MISO [64]. This assembly method is more advantageous in the discovery of low expressed transcripts and alternatively spliced isoforms. However, the success of the assembly is dependent on the quality of the reference sequence being used. Large genomic deletions and mis-assembly of a genome will sequentially propagate into a misassembled or partially assembled transcriptome [54].

A reference-based assembly strategy was extensively being applied in RNA-seq analysis, especially for plant species with an established genome sequence available. *Arabidopsis thaliana* reference genome (TAIR10) has contributed in the transcriptome data alignment and also in the mapping of ChIP-seq data [65,66]. By mapping the RNA-seq reads against Arabidopsis genome (TAIR10), Pajoro et al. (2017) [4] have successfully identified the temperature-induced differentially spliced events in Arabidopsis plants after being exposed to different temperatures. Subsequently, they were able to detect a total of 59,736 regions to be enriched in H3K36me3 after using similar reference genome for the mapping of FASTQ files generated in ChIP-seq. Integration of the RNA-seq and ChIP-seq datasets revealed that the H3K36me3 histone mark was overrepresented in differentially spliced event genes, and reduction in the H3K36me3 mark deposition could affect the temperature-induced alternative splicing.

### 4.4. De Novo Assembly

For species lacking a sequenced genome, de novo assembly of the overlapping reads can be employed using one of the several assemblers, including Trinity [67], SOAPdenovo-Trans [68], and Trans-ABySS [69]. All the de novo assemblers listed above are developed by referring to de Bruijn graph algorithms, which broke the reads into k-mer seeds to construct a unique de Bruijn graph and then parsed into consensus transcripts. Annotation of the consensus transcripts can be achieved by mapping to a genome or alignment to a gene or protein database [70]. There are several general metrics for assessment of the de novo assembled transcriptome quality, such as assembly statistics, contigs statistics, mis-assembly statistics, number of contigs matching with the closest related genome, and number of hybrid transcripts [3]. Typically, de novo assembly of large transcriptome is challenging and requires much higher sequencing depth for better assembly output [54]. Nevertheless, the de novo assembly method still possesses certain merits against reference-guided assembly method in discovery of novel transcripts caused by missing genes or structural variants, identification of transcripts with long introns, and in detection of rare events like trans-splicing and chromosomal rearrangements [71].

### 4.5. Expression Quantification and Normalization for Differential Expression Analysis

Following transcriptome assembly, transcript expression can be quantified by counting the reads mapped to each coding unit including exon, gene, or transcript [72]. For single-end reads, the reads per kilobase of transcript per million mapped reads (RPKM) metric is introduced to remove the feature-length and library-size effects through dividing the number of read counts by both its length and total number of mapped reads. Fragments per kilobase of transcript per million mapped reads (FPKM) is the metric derived from RPKM which is applicable for paired-end reads data and considers a fragment (not reads). Together with transcripts per million (TPM), RPKM and FPKM are the most frequently reported values for transcript abundances in RNA-seq [3,47,70]. Although RPKM/FPKM is a popular choice in place of read count, its value in a sample can be significantly altered by the presence of several highly expressed genes which will “consume” many reads and subsequently underestimated the remaining genes, particularly lowly expressed genes [3]. Wagner et al. (2012) [73] demonstrated that RPKM has the potential to cause inflated statistical significance values due to its inconsistency between samples, which arises from the normalization by the total number of reads. HTSeq (https://pypi.python.org/pypi/HTSeq) is a Python library that contains a stand-alone script *htseq-count* which can count the number of aligned reads mapped to a single gene while discarding multi-mapping reads. These counts can then be used as input data for gene-level quantification using methods such as edgeR or DESeq [74]. The major challenges in read quantification is to quantify multi-mapping reads because of genes with multiple isoforms or close paralogs. In order to address this problem, several algorithms were developed to allow isoform-level quantification. Alternative expression analysis by sequencing (ALEXA-seq) estimates isoform abundances by counting the reads that mapped uniquely to a single isoform, but this method is not suitable for genes lacking unique exons [70]. Alternatively, Cufflinks will quantify isoform abundances by constructing a likelihood function that models the sequencing process to estimate the maximum likelihood that the read maps to an isoform and reports in FPKM or RPKM values [2].

Throughout the RNA-seq experiment, there will be various biases and variances being incorporated which involves intra-sample differences such as differences in length, GC content, or inter-sample differences, for example, differences in sequencing depth, sampling time, and so on [3]. These variations should be eliminated to improve the accuracy of the statistical analysis applied for inferring differential expression. Previous studies have demonstrated that the choice of normalization procedure can impact on the result of differential expression analysis and emphasizes the requirement for normalization [48,75]. Sequencing depth of a sample is one of the major sources of biases in RNA-seq data, therefore trimmed mean of M-values (TMM) and median of ratio approach by assuming most genes are not differentially expressed have been proposed [76]. A comparison study involving seven normalization methods demonstrated that TMM and median of ratio are the two most robust normalization approaches for library size normalization after testing with simulated and real RNA-seq data [77]. The TMM approach has been implemented in R/Bioconductor packages edgeR [78], while the median of ratio approach has been implemented in R/Bioconductor packages DESeq [79], DESeq2 [80], and in Cuffdiff2 [81].

One of the most routinely used analyses conducted using RNA-seq data is to identify differentially expressed genes (DEG) among phenotypes and experimental conditions, and hence, a number of complex statistical methods have been designed to perform this task. Tuxedo suite, a suite of tools for transcript assembly and quantification comprises of Bowtie, TopHat, and Cufflinks packages. TopHat utilizes Bowtie as an alignment “engine” to map millions of RNA-seq reads to the genome and these read alignments serve as input for Cufflinks to produce a transcriptome assembly for each condition. The assembly files are then merged together using the Cuffmerge and fed to Cuffdiff to detect DEGs and genes that are differentially spliced or differentially regulated via promoter switching across multiple conditions. Data generated by Cuffdiff analysis can be visualized and explored with CummeRbund [82]. Additionally, there are several other software tools that support DEG analysis such as edgeR, DESeq2, baySeq [83], and NOIseq [84]. EdgeR software uses an over-dispersed Poisson model to account for biological and technical variations in replicated data, and subsequently applied an empirical Bayes method to alleviate the degree of overdispersion across genes. Lastly, differential expression analysis is performed using either quasi-likelihood (QL) F-test or likelihood ratio test [85]. While DESeq2 is adapted from DESeq with the critical enhancement by incorporating empirical Bayes shrinkage estimators for dispersion and fold change, which facilitates a sound and statistically well-founded differential expression analysis across a wide dynamic of RNA-seq experiments. Besides, through the implementation of shrinkage of fold change on a per-sample basis termed as rlog transformation eases the visualization of differences in heatmap and the application of numerous downstream techniques, including principal component analysis and clustering, in which homoscedastic input data is needed [80].

Costa-Silva et al. (2017) [86] evaluated the impact of six mapping and nine differential expression analysis methodologies on real RNA-seq data and adopted qRT-PCR data as reference. The results indicated that mapping methods have minimal impact on the expression analysis result and highlighted that the DEGs identification method is the main choice for differential expression analysis. Based on the adopted experimental model, NOIseq, DESeq2, and limma + voom [87] are the most balanced DEGs identification software by considering the precision, accuracy, and sensitivity. However, there is no consensus on the best-suited differential analysis method for all circumstances.

### 4.6. Functional Annotation and Pathway Analysis

The final step in a standard transcriptome analysis pipeline is often the interpretation of the gene expression data through gene set enrichment analysis. The analysis would favour the characterization of the functional annotation of the listed DEGs and their associated biological pathways or molecular function in order to infer biological insights from these genes. Publicly available resources, such as Gene Ontology [88] and DAVID [89], containing annotation databases of gene products for most model species are commonly used for gene annotation purposes and also would allow identification of functional information across orthologs [47]. On the other side, multiple listed DEGs may have interactions with each other and be involved in certain biological pathways. KEGG (Kyoto encyclopedia of genes and genomes) pathway database provides a valuable resource for investigating significantly enriched biological pathways associated with the listed DEGs [90]. MapMan4 [91], which is the latest version of MapMan framework coupled with the revised Mercator4 online tool, provides another option for protein classification and annotation task of any land plant. With the triple increased total number of bin categories, MapMan4 has been improved to perform more precise protein descriptions for all assignments through a leaf node category. The prediction of protein–protein interaction network would facilitate the understanding of cellular processes and annotation of structural and functional properties of proteins. This analysis can be performed using the STRING database (http://string-db.org/), which is a web resource of known and computational predicted protein interactions [92]. During the de novo transcriptome reconstruction, there will be a number of unknown transcripts being discovered, and Blast2GO can be used for homologous gene identification through GenBank BLAST or InterProScan and assigning gene ontology terms to each locus [93].

## 5. ChIP-seq Workflow (Wet Laboratory)

ChIP-seq assay is a powerful tool used to determine nuclear protein interactions with DNA that is usually applied in the context of disease diagnostics, gene expression, and cell differentiation in animal systems for personalized medicine development. Plant scientists have now adopted the technique to better understand various in vivo epigenetics changes and discover genes expressed in a certain biotic/abiotic stress response (that is protein–DNA interaction) in plant systems. The encyclopedia of DNA elements (ENCODE) is now the largest database of sequencing-based techniques, including ChIP-seq. The database has a massive amount of information limited to four different animal species, namely; human, mouse, worm, and fly. ENCODE encodes ChIP-seq overview information covering experimental design to data analysis and contains some published standards to achieve each step in ChIP-seq analysis. Plant researchers can employ the same ENCODE standards. For instance, the ENCODE manual [94] reports step-by-step methods for primary and secondary characterization of protein/antibody, which can also be applicable to plants. Western blot and immunoprecipitation are amongst the primary methods, while secondary methods are subordinates to the primary methods and they use previously characterized antibodies for ChIP-seq, epitope-tagged expression pattern, motif analysis, etc.

**Figure 2:**
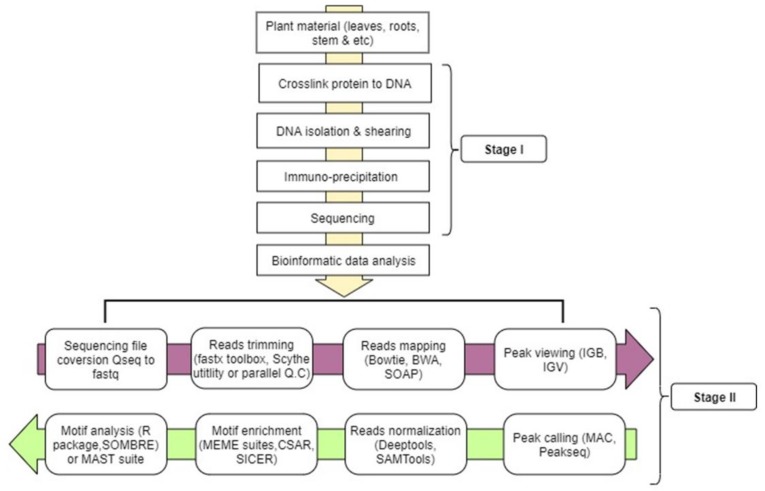
Basic steps involved in ChIP-seq: stage I starts from crosslinking to sequencing and stage II involves steps for gene mining. Sequenced file Qseq are converted to fastq format using fastx tools, reads undergo trimming and filtering using Scythe utility or parallel Q.C, then reads alignment using Bowtie or Burrow Wheelers alignment (BWA), matched reads viewing is aided by integrative genome browser (IGB). Peaks are called using any available software like MAC/Peakseq, reads are normalized by removing duplicate reads and searching for tag densities in a window of reads per kilobase per million reads (RPKM) around the reference peak, mostly 1 kb upstream of transcription start site (TSS) to transcription end site (TES), with SAMTools, motif search using MEME suites, and finally predicts gene through MAST suite or R statistic package SOMBRE with the aid of GO and transcription factor databases like JASPAR.

For plants, the ChIP-seq protocol usually takes about 3–7 days to completion, starting from nuclei extraction to immunoprecipitation. There might be changes in some steps which are geared towards reducing time consumption and simplifying the tedious nature of the technique. In this section, major steps of ChIP-seq will be reviewed starting from formaldehyde fixing of plant sample, chromatin isolation, and to data analysis (as shown on Figure 2).

### 5.1. Crosslinking in Plant Samples

Formaldehyde is a small (2Å) dipolar compound that can entrap protein–protein and protein–DNA complexes in vivo. Its small size makes it the best candidate for capturing macromolecular interaction that are close to one another [95]. Its carbon atom plays a role as a nucleophilic center. Amino and imino functional groups of DNA (adenine and cytosine) and of some amino acids (arginine, histidine, and lysine) readily react with formaldehyde to form a Schiff base intermediate. This can also react to another amino group to form the final crosslinked protein–DNA complex [96].

**Figure 3:**
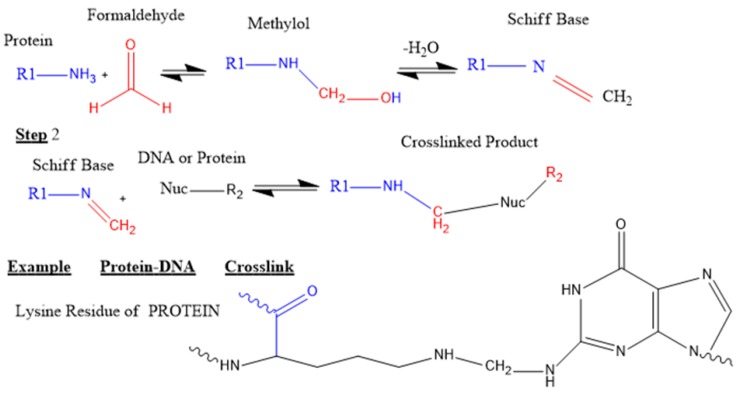
Chemical reactions of protein–DNA crosslinking by formaldehyde: Crosslinking of protein–DNA by formaldehyde occurs in two steps. Firstly, a strong nucleophile, commonly a lysine є-amino group from a protein, reacts with formaldehyde to form a methylol intermediate which will lose water to give a Schiff base (an imine). Secondly, the Schiff base reacts with another nucleophile amine of a DNA to generate a crosslinked product. The latter nucleophile might also be from another protein or the same protein as the first nucleophile. All the reactions in this stoichiometric process are reversible. Modified from Hoffman et al. (2015) [97].

**Figure 4:**
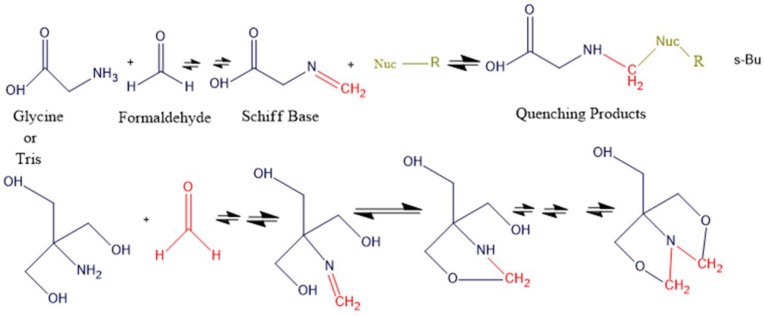
Glycine and Tris quenching reactions of formaldehyde: The chemical reactions are like those shown in Figure 3 above with the amino group of glycine or Tris serving as the principal nucleophile. The Schiff base formed from glycine is not necessary to react with a second nucleophile, but regardless, the crosslinking between protein–DNA will be quenched. The Tris molecule has another available nucleophile (hydroxyl group) that creates stable intramolecular penta-membered rings. Tris can also react with two molecules of formaldehyde, resulting in the last product shown. The tendency of Tris forming some stable intramolecular products allows it to search for formaldehyde from other molecules and thus enable crosslink reversal. Here, mint green color represents DNA/protein. Reconstructed from Hoffman et al. (2015) [97].

Formaldehyde is now the crosslinking agent of choice in ChIP-seq protein–DNA binding due to its robustness, reversibility, and less hazardous nature compared to use of ultraviolet (UV) radiation as a cross linker method [97,98]. For example, Haring et al. (2007) [35] used 3% formaldehyde to crosslink protein to DNA region in Maize (*Zea mays*) while subsequent publications used 1% formaldehyde in *Arabidopsis thaliana* [99,100]. Hoffman et al. (2015) [97] has reviewed formaldehyde binding chemistry and showed a two-stage stoichiometry mechanism of crosslinking protein–DNA with formaldehyde and quenching with glycine. Figure 3 and Figure 4 show the two-step forward chemical reactions of crosslinking and quenching of excess crosslinking agent respectively. Reversal of protein–DNA crosslinking is typically achieved by heating (usually 65 °C) in the presence of high salt concentrations (example 5 M NaCl and 20% SDS) [35,101] or treating with proteinase K at 37 °C [100].

The reversal of protein–DNA crosslinking is normally achieved by heating (at 65 °C) in the presence of high salt concentration (example: 5 M NaCl and 20% SDS) [99,102] or by treating with proteinase K at 37 °C [100].

### 5.2. Chromatin Isolation

The method of nuclei isolation is dependent on the source of plant sample and quantity (required mostly 1 to 5 g). For example, whether it is a high phenolic and carbohydrate content like oil palm and Jatropha, which may need higher concentration or a longer treatment period with cell wall degradation components like Triton x-100 detergent in nuclei extraction buffer [103], and if physical shearing or if the sample is from a delicate plant such as Arabidopsis, in which nuclei can be isolated with mild extraction buffer. A protocol developed by Saleh et al. (2008) [99], also by Kaufmann et al. (2010) [100] and many more, explains the laboratory procedure for chromatin extraction. On the other hand, DNA shearing optimization is quite similar across different plant biology laboratories and it is achieved by sonicating the nuclei in a probe sonicator, or water bath ultrasonicator five times (more or less), 30 s ON and one minute OFF on ice (keep the whole step in cold conditions) until a desired DNA fragment size is achieved, which should be within 100–800 bp [35,100,104]. Shearing can also be achieved using an endo-exonuclease MNase [105], but random shearing is mostly not achieved using MNase due to the present of specific cut sites, and this makes sonication the most preferred shearing method since its DNA defragmentation is random.

Immunoprecipitation is a pulldown assay which involves an antibody designed against protein of interest or against a tagged DNA fragment (tags like FLAG, GFP, yellow fluorescent protein (YFP)) coupled with protein of interest, which is used to pull all DNA bound to the protein tag [98]. Conventionally, chromatin is incubated with 1 to 5 μg antibody overnight [106,107,108] to appropriately pull down all DNA fragments.

Obviously, ChIP-seq is a difficult immunological assay in plants. The major problems plant scientists are facing includes cell wall complexity of plant cell that requires vigorous disruption to avoid sample loss; high level of polysaccharides and phenolic compounds in plant tissues may be a problem for PCR amplification prior to library preparation; ChIP-grade antibodies selection in plants is limited, and as a result of that, investigators will have to take several months to generate epitope-tagged transgenic lines before ChIP-seq experiments [34]. These problems are not yet solved with the plant researchers, but significant contributions have been made to address some part of the problems such as high DNA recovery [105,109] and production of customized protein-specific antibody [110], which takes a similar period of time as transgenic epitope-tagged antibodies do.

### 5.3. ChIPped-DNA Purification

After immunoprecipitation, antibody ChIPped isolated DNA is followed by a purification step preceded by de-crosslinking. There are several methods for ChIPped-DNA purification, amongst which Zhong et al. (2017) [105] compared ten commercial kits and observed that phenol-chloroform (Invitrogen; PC) method gives the best DNA recovery. Interestingly, they found PC to yield the best DNA recovery. DNA recovery is important because ChIP usually gives insufficient amount of DNA for library construction and qPCR, and sometimes, PCR of the recovering DNA is required.

### 5.4. Library Construction

Sequencing library construction is the last stage of bench work for ChIP-seq assay and the subsequent steps will only be in silico. Library preparation is often carried out with the use of commercially available kits. From the recent literature, researchers often use kits such as Illumina TruSeq library protocol [40,111], NEBNext ChIP-seq library preparation reagent for Illumina kit (New England Biolab) [40,112], ThruPLEX DNA-Seq kit (Takara Bio USA) [65], and Ovation ultralow library kit (NuGEN Tech, San Carlos, CA, USA) [113,114]. Usually, for sequencing, reads preparation are either single-ended or paired-ended and can also be both. From the names, sequencing one end or both ends of DNA fragment is referred to as single-end tags (SET) and paired-end tags (PET), respectively. SET is the commonly used option while PET is more precise, less ambiguous in genome alignment, and typically used for repetitive fragments of genome [115]. After all, reads sequencing is commonly supplied by HiSeq 2000–3500 Illumina machine (San Diego, CA. US).

## 6. ChIP-seq Workflow (Data Analysis)

ChIP-sequencing data contain millions of short nucleotide sequences based on sequencing depth. The depth of the sequence depends on the organism’s genome size, the probable binding site’s size, and frequencies [116]. For example, 43 million reads are adequate for studying TFs involved in stress response mediated by jasmonic acid (JA) signaling pathway in rice [39] and approximately 25 million reads for analyzing maize endosperm development [117]. To get a reliable result, an ENCODE consortium standard of using two independent biological controls should be followed. This will help to assess replicates’ agreement and threshold with the use of irreproducible discovery rate (IDR) [94]. Complexity of ChIP-seq libraries is linked to several factors such as antibody quality, over-cross-linking, amount of material, sonication, or over-amplification by PCR. Hence, the last factor can be corrected by systematic identification and removal of redundant reads, which is implemented in many peak callers because it may improve the specificity of PCR [116].

Large ChIP-seq data output analyses usually employ a stage-wise bioinformatic software pipeline and webtools for proper data interpretation and visualization. The steps include sequence alignment to a reference genome (mapping), peak calling, motif discovery, and interpretation [118], as shown in Figure 2. There are several reviews on ChIP-seq computational analysis encompassing reads quality control to TF motif discovery [119,120].

### 6.1. Reads Mapping

Alignment of ChIP-seq reads signal in the genome region has three categories: small point source base pairs coverage (known as punctate region with few kilobases) of localized signals, such as transcription factor and broad region of several kilobases that covers a large epigenetic domain like H3K36me3, and a mixed region which covers both transcription site on upstream part of a gene [120,121] and within the downstream part of a gene like RNA polymerase [122,123]. Nevertheless, there is a need for a complex normalization procedure for significant variation distribution coverage among samples, not only relying on the sequencing depth but similarly on library preparation methodological differences and sample disparity [124], as well as chromatin condition of the samples. There are a few ChIP-seq analysis normalizations methods in the public domain to provide identical coverage distribution across samples [125]. A peak is considered if its number of readings is higher than a predetermined cut-off value or if a minimum enrichment value equated to the background signal, is frequently in a genome through a sliding window. Many peak calling algorithms give an approximate calculation of a *p* value for called peaks, height of the peaks, and/or background rank peaks enrichment and a FDR to provide peak list [126]. PeakSeq mapping analysis technique [123] compares control sample and IP threshold factor coverages for two linear regression, a quantile normalization technique proposed by [127], which uses statistical moments for the normalization process [125]. Bowtie [56] indexes a reference genome based on the Burrows-Wheeler transform (BWT) [128] and FM index [129]. Besides these, there are many sequence aligners, but the most widely used are SOAP2 [130], BWA [57], Hisat2 [131], and DANPOS2 suite with Dpeak [132]. Bowtie seems to be the most preferred [110,113,133,134] based on the literature, while DANPOS2 [132] is the least preferred. Likewise, numerous software packages are used for peak calling, but the most popular peak caller is MAC [135], as reported in several publications [136,137,138].

### 6.2. Enrichment of Genomic Region

In order to determine TF binding site on a plant’s genome, special web-based tools and software packages are designed to help with motif finding analyses. Some of these tools are based on various kinds of algorithms which are statistically dependent. According to the most recent ChIP-seq reports, the following motif enrichment tools are commonly used for genome enrichment: MEME [139], MEME/MAST suite [140], and the most repetitive in literature [38,40,112]; DREME [141], RSAT [142], CSAR Cisgenome [125], SICER package [143], and BEDTools [144]. Table 1 provides a summary of the trend of some ChIP-seq publications from 2014 to 2019 series, highlighting the field of research and emphasizing the type of antibody used.

## 7. Genome-Wide Identification of Transcription Factor Co-Regulated Genes by RNA-seq and ChIP-seq

Shamimuzzaman and Vodkin (2013) [38] were interested to understand early seedling developmental stages in soya bean. They classified the stages into 7 (from pre-emerging hypocotyls to fully grown cotyledons above the ground) and performed RNA-Seq on 7 libraries generated from the different stages. The RNA-seq generated 78,773 mapped reads using ultrafast bowtie, allowing three mismatches. Reads normalization was followed using RPKM and DESeq package to identify the DEGs between developmental stage 3 and 6 at *p*-value < 0.05. Two TFs, NAC and YABBY, which showed promising expression levels throughout the different stages, were chosen. Consequently, NAC and YABBY antibodies were used to perform ChIP-seq using pooled cotyledons from stage 4 (yellow-green cotyledons 30–35 mm) and 5 (yellow-green cotyledons; starts of primary roots) which is a physiological transition stage between yellow food reserve to a photosynthetic green stage in order to identify their genome-wide binding sites and their co-regulated genes. ChIP-seq data was first aligned using Bowtie to generate 34 million reads and 86 million reads for NAC and YABBY, respectively, at *p*-value < 0.05, and subsequently MACS was used to call significant peaks, which were 8246 and 18,064 peaks, respectively, for the two TF at *p*-value = 1.0 × 10^−5^**.** Gene location were identified from the soya bean gene annotation using a custom-made Python program. In the promoter, there were 1526 and 974 peaks for NAC and YABBY, respectively. These two TFs play an important role in regulating developmental processes and the sequence similarity analysis between RNA-seq, and NAC and YABBY TFs ChIP-seq data showed 72 genes to be potentially regulated by the NAC and 96 genes by the YABBY.

Opaque2 (O2) TF is involved in maize endosperm development and its mutation *o2* confers better nutrition with 70% higher lysine content (quality protein maize; QPM) than in wild type maize kernels, which were studied. However, the mutant plant exhibited some pleiotropic biological effects that lowers its agronomic quality. Li et al. (2015) [117] performed RNA-seq on both wild-type (WT) O2 and mutant *o2* endosperms. Fifty-five million reads were uniquely mapped to B73 maize genome sequence using TopHat and further normalized as fragments per kilobase of exon per million fragments mapped at *p*-value < 0.05 using MACS, 52,601 genes were found to be transcribed in both O2 and *o2*. Further analysis narrowed the genes to 3070 in O2 and 6613 genes in *o2*. At last, 1605 genes mRNA steady levels were affected by O2: 767 upregulated in O2 and 838 upregulated in endosperm deficient in O2 function. On the other hand, ChIP-seq assay was performed using O2 custom antibody on wild-type plants, and 15 million reads were specifically mapped using Bowtie2 aligner, while 1686 peaks were mapped using MACS at q-value < 0.05 by comparing O2 and IgG ChIP outputs based on poisson distribution. RNA-seq revealed 1605 DEGs between wild-type and mutant endosperm while ChIP-seq identified 39 genes as O2 putative target. Thirty-five of them were down-regulated in *o2* RNA-seq, while four were upregulated. But none of the DEGs found in *o2* were identified as O2 putative binding target in ChIP-seq, suggesting the potential involvement of non-coding RNA as downstream targets. Combination of the RNA-Seq and ChIP-Seq results had demonstrated the roles of O2 as a central regulator of multiple metabolic pathways related to anabolic functions during maize endosperm development.

Jasmonic acid (JA) mediates activation of plant resistance against insect attack (wounding) and necrotrophic pathogens. MYC2 is a basic helix-loop-helix (bHLH) TF which plays a significant role in orchestrating JA-mediated expression of defense genes. To understand the role of MYC2 TF in tomato, Du et al. (2017) [40] performed RNA-seq on mutant (MYC2-RNAi) and wild-type treated with or without methyl jasmonate (MeJA); wild-type wounded and no wound wild-type. Bowtie2, HISAT2, and TopHat2 were used to align sequencing output to tomato genome SL2.50. Expression levels were determined using eXpress [158] for calculating gene expression levels in all biological replicates at FDR-adjusted *p*-value < 0.05. DESeq2 was used for mRNA levels quantitation at *p*-value < 0.05. Pairwise comparisons of RNA-seq data recognized 6544 genes that were DEGs between treatments (with and without MeJA). Two thousand, five hundred and sixty-seven genes showed significant expression differences between untreated mutant and wild-type and 3058 genes showed significant expression differences between mutant treated and wild-type. Additional analyses of these 3058 genes revealed about 40% (2558 from 6544) of the JA-regulated genes also regulated by MYC2. Whereas, ChIP-seq was performed on MYC2-GFP transgenic plants either subjected to MeJA or wounding treatment. Sequence alignment using Bowtie2 and mapping using MACS at q-value < 0.05 were performed. BEDTools with default parameters were used to identify peaks within genic regions and a total of 12–18 thousand putative MYC2 binding peaks from the two biological replicates were identified. The replicates shared 7594 peaks agreement and further overlapped to identify 3389 MYC2-targeted genes. Comparison of ChIP-seq 3389 MYC2-targeted JA-responsive genes (MTJA) and pairwise comparison of RNA-seq data output identified 2258 genes are coregulated by MYC2 and JA. After comparing these two data sets, 655 genes were found to overlap for MYC2-targeted JA-responsive genes (MTJA). The study also identified a group of MYC2-targeted TFs that may have a direct role in regulating the JA-induced transcription of late defense genes. Altogether, it was proposed that MYC2 and the MYC2-targeted TFs form a hierarchical transcriptional cascade during JA-mediated plant immunity responsible for the initiation and amplification of the transcriptional output.

Gibberellic acid (GA) normally promotes plant growth by targeting the destruction of DELLA proteins [158]. Wheat GRV DELLAs mutant [159] is resistant against GA destruction, whereas the rice GRV mutant sd1 allele diminishes bioactive GA abundance [160,161], leading to the accumulation of DELLA protein SLR1. This confers semi-dwarfism and results in yield-reducing lodging. Lodging resistance by GRV increases nitrogen insensitivity associated with nitrogen-use efficiency. Growth regulating factor 4 (GRF4) semi-dominantly increases nitrogen (NH4^+^) uptake rates and assimilation while SLR1 inhibits these processes. Li et al. (2018) [152] carried out a study through combined Omics (RNA-seq and ChIP-seq) to understand this process. Firstly, RNA sequencing was performed using BGISEQ-500 platform to produce 24 million clean reads mapped to Nipponbare reference genome with HISAT/Bowtie2 tools. The reads were normalized and FPKM was calculated using RSEM software [159]. DEGs were identified using FDR < 0.01 and absolute log_2_ ratio ≥ 2 [160]. Four thousand, two hundred and forty-one DEGs were identified between mutant (loss of function) and WT plants, while 4753 DEGs were accumulated between overexpression line and WT. Six hundred and forty-two genes were identified by RNA-seq to be upregulated by GRF4 in a rice overexpressing GRF4 variety and downregulated by SLR1 in sd1 mutant variety. Quantitative reverse transcription PCR (RT-qPCR) shows high abundance of root mRNAs for NH4^+^ uptake transporters (AMT1.1 and AMT1.2). For the ChIP-seq assay, after BGISEQ-500 sequencing output were mapped to Nipponbare reference genome using SOAP aligner, MACS was used to call potential binding peaks. To define genomic location type, peak summit was used to overlap 100 bp around the top of the peak summit, which was then subjected to DREME motif analysis. Two loci density were drawn using density plot tool in R 3.0 after a likelihood ratio score was considered for each motif in each peak using the basic principles of Bayesian classifier [136]. ChIP-seq revealed potential GRF4 target-recognition sites, with a predominant upstream GGCGGC binding motif common to many nitrogen-metabolism gene promoters. Enriched ChIP-seq DNA through ChIP–PCR confirmed the RT-qPCR finding of GRF4 ammonium transporter AMT1.1 with a putative GCGG- promoter motif.

Albihlal et al. (2018) [39] studied resistance to environmental stress and reproductive fitness (seed yield) regulated by heat shock transcription factor A1b (HSFA1b) protein in Arabidopsis thaliana. To unravel the function of HSFA1b, they surveyed its ChIP-seq target and its significance on its RNA-seq transcriptome of wild type under heat stress (HS) and non-stress (NS), and in transgenic HSFA1b-overexpressing plants under NS. RNA-seq analysis workflow started from sequencing outputs from Illumina HiSeq2000. Reads were mapped to Arabidopsis transcriptome GSNAP (allowing five mismatches). Transcript assembly and DEGs analyses were followed using Cufflinks and Cuffdiff [82] at q-value ≤ 0.05. For wild-type treatment under NS and HS, 7137 DEGs responded to HS: 721 were HSFA1b-bound genes. These bound genes were prevalent in downstream of protein-coding genes suggesting binding to genomic regions or near cis natural long non-coding (*cis*NAT) RNA genes. RNA-seq from HSFA1b-RFP overexpression lines under NS revealed 3306 protein-coding genes showing differential expression when compared with NS WT, and 72% of them were differentially expressed in HS WT. After a Pearson correlation between NS 35S:HSFA1b and both NS (r = 0.92) and HS WT (r = 0.88), heat shock proteins expression levels in 35S:HSFA1b NS plants were found to be intermediate to WT NS and HS plants. Further analyses found a total of 952 HSFA1b-target DEGs and at least 85 of them were developmentally associated and found bound mainly under NS. In addition, 480 natural antisense non-coding RNA (cisNAT) genes bound by HSFA1b were identified, suggesting an additional mode of indirect regulation. On the ChIP-seq analysis, normalized peaks were called with MACS tool and *k*-means clustering analysis of ChIP-seq signals on HSFA1b bound genes and density maps were generated with seqMINER [161]. GO analysis of target features was performed with a singular enrichment analysis (SEA) tool in the AgriGO database [162]. HSFA1b bound region sequences motif were de novo identified by using MEME with *p-*value < 0.0001 and passed through Cistrome atlas database [163]. ChIP-seq identified 1083 and 709 HSFA1b-bound regions under NS and HS, respectively, consisting of 1207 HSFA1b target genes. *K*-means cluster analysis of binding regions identified three groups: specific to NS (group I), common to NS and HS (group II), and unique to HS (group III). After a deep analysis of binding regions in gene annotation features, HSFA1b was found to be preferentially targeted within and downstream of genes in group I (54%) while it was 30% for group II and III genes. NP:HSFA1b ChIP-seq data under HS and NS conditions intersection with the DEGs from 35S:HSFA1b compared with NS WT plants revealed 1821 genes in WT HS plants and in 35S:HSFA1b NS plants that were not bound by HSFA1b. These were designated to regulate HSFA1b indirectly, of which 281 genes were associated with plant development. It was also found that the HSFA1b does not only target the heat shock elements, but also the MADS box, LEAFY, and G-Box promoter motifs. Thus, this suggested that HSFA1b transduces environmental cues to many stress tolerance and developmental genes to enable continuous growth and developmental adjustment by plants in a varying environment under diverse environmental factors.

JA is a plant hormone involved in different plant biological processes such as plant growth, seed germination, response to water stress, wounding, and pathogen attack [40,164]. TF basic region/leucine zipper (bZIP) belongs to a diverse superfamily of TFs divided into 13 groups in Arabidopsis. VIP1, a member of bZIP group I TF, is a bridge between nuclear importin α and VirE which facilitates transport of *Agrobacterium* T-DNA strand into plant nucleus [165]. In addition to its role in Agrobacterium-mediated transformation, it functions in *Botrytis* attack, salt stress, and ABA responses [166,167]. Liu et al. (2019) [39] studied bZIP TF activity using multi-Omics strategy in rice. RNA sequencing output was generated using HiSeq3000 and mapped to *Oryza sativa* reference genome (RGAP v. 7.0), performed on OsbZIP81.1ox and WT ZH11. Gene expression levels and identification of DEGs were carried out using RPKM and edgeR, respectively. Five thousand, one hundred and forty-three DEGs (in OsbZIP81.1ox versus ZH11) and 5002 DEGsOsbZIP81.2ox versus ZH11) were identified. ChIP-seq analysis on OsbZIP81.1 under normal condition yielded 43 million reads after SOAP2 alignment and unique mapping with MACS. These reads were subjected to motif analysis using MEME-ChIP. They carefully analyzed the combined ChIP-seq and RNA-seq data of rice OsZIP81.1 and found 7 genes that were enriched in JA signaling pathway from 1332 genes that were identified. For binding motif discovery by ChIP-seq, they found 15 probable motifs which were referred to as Oryza VIP1 response element (OVRE) GCTG, which are closely related to the Arabidopsis VRE.

## 8. Third Generation Sequencing

The progress in NGS development has enabled researchers to study and understand the complex world of microorganisms, plants, and animals from broader and deeper perspectives. In the third-generation sequencing technology, platforms were designed to address the limitation in obtaining an effective read coverage, especially in the short read lengths, which are poorly suited for particular biological problems, including assembly and determination of complex genomic regions, gene isoform, and DNA methylation detection [168]. This is due to inherent limitations of the short-read technologies such as GC bias and problems associated with mapping to repetitive regions, differentiating paralogous sequences, and phasing alleles [169]. Long-read/third-generation sequencing technologies revolutionised genomics research as they enable genomes and transcriptomes to be analysed at an unprecedented resolution. It allows direct native DNA and full-length transcript sequencing without requiring sequence assembly. Oxford nanopore and pacific biosciences offer long sequence read technologies commercially. Both use single-molecule sequencing, but with contrasting detection methods based on nanopores and optical detection, respectively. Both provide exceptionally long read lengths, up to greater than 20 kb. These platforms allow sequencing/assembly of repetitive elements, direct variant phasing, and determination of epigenetic modifications [169].

The pacific biosciences RS platform was first released in 2010. It uses hairpin adapters ligated on either end of a DNA molecule to be sequenced, generating capped templates referred to as single-molecule real-time (SMRTbells) [169]. SMRT sequencing is a sequencing-by-synthesis technology based on real-time imaging of fluorescently tagged nucleotides that are incorporated as complementary strand is synthesized along individual DNA template [170]. DNA modifications such as methylation are detected based on the kinetic variation obtained from the light-pulse. The technology allows generation of full-length cDNA sequences without the need for assembly and characterization of transcript isoforms within targeted genes or across an entire transcriptome. It uses a DNA polymerase to drive the reaction and the sequencing reaction ends when the template and polymerase dissociate [171]. The average read length is about 3000 bp, but some may reach 20,000 bp or even longer [172].

NGS methods tend to lose information found in DNA and RNA due to the short-copied reads and the inability to retain modifications. The Oxford nanopore technologies methods that were first commercialised in 2014 can overcome these limitations through direct DNA and native poly(A) RNA sequencing strategy. It does not involve any fragmentation and amplification steps, which are potential source of bias faced in the NGS technology. The sequencing adapter contains the motor protein, an enzyme controlling the passage of the nucleic acid through the nanopore. Read length is directly proportional to the length of RNA and DNA being prepared. Nucleic acid bases determination based on changes in electrical conductivity that are generated as the RNA/DNA strand passes through a biological pore does not require chemical tagging of nucleotides. Sequence data information is produced in real-time, enabling direct data analysis and processing [173]. The long-read nanopore RNA sequencing enables precision characterisation of complete DNA and full-native RNA sequences facilitating sequence assembly and mapping. It enables unambiguous determination of transcript isoforms, giving a true reflection of gene expression with high sensitivity down to single cell level [174].

## 9. Conclusions and Future Prospects

The identification of DEGs is now widely available via high throughput analysis of transcriptomes. However, the quality of data generated is highly dependent on the experimental design, quality of RNA, and sequencing depth. The information generated will not always provide enough evidence of the specific role of transcription factor, the master regulator at the transcriptional level in regulating certain biological pathway or physiological process, as it only enables inferences based on co-expression of genes. In contrast, genome-wide identification of transcription factors of interest or novel transcription factors through RNA-seq can result in a more in-depth understanding of transcriptional networks associated with these transcription factors and their regulons when followed with ChIP-seq analysis. The ChIP-seq technique can provide valuable information about transcriptional regulation based on transcription factor binding to target DNA promoter motifs for coordinating transcriptional regulation in response to environmental cues, while RNA-seq alone does not provide complete information. However, a combination of these technologies opens up new prospects to better elucidate more comprehensive gene regulatory networks. This approach provides a better explanation of gene regulatory networks [117] and opportunities to explore uncharacterized genes (new genes in a treatment). The approach can give more insights in TFs and the networks they regulate, hence allowing functional study on prospective TFs found in this approach. Although integrating data assembly tools from both ChIP-seq and RNA-seq data like Partek [175] and BETA [176] could be interesting, unfortunately this approach needs to develop the full options of parameter inputs or algorithms required for each method [126]. Advances in the development of new peak calling algorithms, as briefly described in this paper, are providing more reliability and precision concerning the genes detected from high throughput sequencing data [126]. Specifically, in Salmon and Sailfish RNA-seq data analysis, pseudo-counting was introduced at the read counting stage to avoid large negative log transformed values and arithmetic error [177]. This, in combination with third generation sequencing with improved accuracy of sequence assemblies and the discovery of sequence variants, will enhance the potential of combined ChIP-seq and RNA-seq applications that can better describe the functionalities of complex plant genomes and their regulatory networks.

One way to alleviate the problem of TF-specific antibody development is to use clustered regularly interspaced short palindromic repeat associated-Cas9 (CRISPR-Cas9) for epitope tagging. CETCh-seq is designed to tag DNA-binding proteins and subsequently results in using a standard Cas9 antibody [178,179,180]. This will lead to the creation of individual TF maps and the maps between TF can cross-talk to give a network of TF maps, a new prospect offered by this technology. Likewise, ChIP-seq DNA motifs can be verified using CRISPR single-guide RNA (sgRNA) [181] to target the DNA motif [182]. This will pave a way for precision DNA motif discovery with CRISPR double checking.

Finally, the prime goal for this kind of multi-Omics approach is to comprehend big chunk of data generated from genomic research, which can be a source of confusion and wrong inferences. Combining RNA-seq and ChIP-seq output is like following a reductionist approach from millions of reads of RNA-seq DEGs and thousands of ChIP-seq mapped reads to a few functional TFs binding motif(s). Therefore, the plant community can understand how different experimental studies were designed and approached using multi-Omics technique, and importantly, the analysis part where two-way ANOVA and Python script and R software were employed to aid in clear understanding of data [38,40,153].

## Figures and Tables

**Table 1:** A summarized ChIP-seq research findings highlighting the kind of research and type of antibody used.

Application	Findings	Antibody Type	Refernce
Abiotic factor	Abscisic acid stress (ASR5) a TF binds Sensitive To Aluminum Rhizotoxicity 1 (STAR1) promoter in other to positively response against Aluminum stress in rice.	Anti-ASR5	Arenhart et al. (2014) [145]
Developmental + immunity	ROS and defense responsive genes were repressed by HBI1 indicating defense function of HBI1 and also indirectly plays a role in repressing growth through activation of growth-inhibiting HLH genes. HBI1 was also learned to bind to positive activators brassinosteroids function.	Anti-YFP	Fan et al. (2014) [112]
Genetics	In genetic imprinting, some subsets of genes are expressed according to their parental origin. Paternally expressed genes (PEGs) were associated to maternal-specific H3K27me3.	Anti-H3K27me3	Zhang et al. (2014) [146]
Abiotic + developmental	Abscisic, Stress, Ripening (ASR1) from tomato is upregulated in drought stress which acts primarily in the cell wall.	Anti-ASR1	Ricardi et al. (2014) [134]
Developmental	SQUAMOSA Promoter Binding Protein-Like3 (SPL3) bind GTAC motif of phosphate starvation responsive gene promoters like *PLDZ2, miR399f,* and *Pht1;5*.	Anti-HA	Lei et al. (2016) [147]
Developmental	Combinatorial action affect MADS-box transcription factors FLC and SVP in flowering shows gibberellins’ processing genes.	Anti-GFP	Mateos et al. (2015) [137]
Genetics	In circadian clock of Maize hybrids, expression of morning-phased genes from binding with ZmCCA1 encourages growth vigor and photosynthesis.	Anti-CCA1	Ko et al. (2016) [110]
Photosynthesis	Discovers E-box variant binding motif for Phytochrome interacting factor 4/5 (PIF4 and PIF5) in Cryptochromes (CRYs) during exposure to low blue light and CRY2 association with PIF4/5.	Anti-HA	Pedmale et al. (2016) [148]
Cellular	Shows chromatin domain organization at the nuclei periphery of Arabidopsis. The domain is a clear translation of a repressed environment that contains jumping genes, heterochromatic marks and silenced coding genes.	Anti-GFP	Bi et al. (2017) [149]
Immunity	Using both ChIP-seq and RNA-seq, 655 MYC2 binding were identified in response to Jasmonic acid genes. Also found MYC2 TFs that function in late defense stage.	Anti-GFP	Du et al. (2017) [40]
Immunity	After flagellin (flg22) treatment, HD2B targets chromatin were hyperacetylated responsible in plant immune defense and phosphorylation while hypoacetylated marks function in metabolic regulation, plastid organization, and chloroplast.	Anti-GFP	Latrasse et al. (2017) [133]
Biochemical	A zinc finger TF of rice ZFP36 inhibits ROS production by binding to ascorbate peroxidase known to have specificity to hydrogen peroxide.	Anti-ZFP36	Huang et al. (2018) [150]
Developmental	Maize GIF in leaves and stems promotes meristematic determinacy and shoot architecture. ChIP-seq has found several GIF1 targets including mostly some transcriptional regulators like UB3, ZMPLATZ5, ZMARR7, bHLH and MYB family members.	Anti-GFP	Zhang et al. (2018) [114]
Developmental	FRUITFULL (FUL), a TF that directly repressed APETALA2 expression promotes meristem arrest and maintains the sequential expression of meristem maintenance factor WUSCHEL.	Anti-GFP	Balanzà et al. (2018) [151]
Abiotic factor	bZIP10 found to be active in Zinc regulation in *Brachypodium* which relate to oxidative stress and a motif homologous to Arabidopsis was found TGDCGACA.	Anti-GFP	Martin et al. (2018) [152]
Abiotic factor	Growth-Regulating Factor 4 (GRF4) TF co-interacts with growth inhibition regulator DELLA to regulate carbon, nitrogen metabolism and growth.	Anti-FLAG	Li et al. (2018) [153]
Abiotic factor	Rice OsTF1L mapped drought related stress and lignin biosynthesis genes.	Anti-MYC and anti-RNA Pol II	Bang et al. (2019) [154]
Epigenetics	Genome-wide ADCP1 is linked with chromosome enrichment site (pericentrome) and co-localization with H3K9me2.	Anti-GFP	Zhao et al. (2019) [155]
General	GmBZL3 is a brassinesteroids signaling molecule cross talking with many pathways like disease-related, immunity response pathways and hormone signaling.	GmBZL3 antibody	Song et al. (2019) [156]
Developmental	A Leucine zipper domain TF FD plays a crucial role in floral transition.	Anti-GFP	Collani et al. (2019) [157]
General	Found new Oryza VIP1 response element (OVRE) cis-element in abiotic and biotic responses.	Anti-FLAG	Liu et al. (2019) [39]

## Abbreviations

NGS	Next Generation Sequencing
RNA-seq	RNA Sequencing
RIN	RNA Integrity Number
rRNA	ribosomal RNA
mRNA	micro RNA
siRNA	small interfering RNA
piRNA	piwi-interacting RNA
cDNA	complementary DNA
dUTPs	deoxy-UTPs
UDG	Uracil-N-Glycosylase
SBS	Sequencing by synthesis
BAM	Binary of SAM
IGV	Integrative Genomic Viewer
RPKM	Reads per kilobase of transcript per million mapped reads
FPKM	Fragments per kilobase of transcript per million mapped reads
TPM	Transcripts per million
TMM	Trimmed mean of M-values
DEG	Differentially Expressed Gene
ChIP-seq	Chromatin Immunoprecipitation and Sequencing
UV	Ultraviolet
ChIP qPCR	ChIP quantitative realtime PCR
ENCODE	Encyclopedia of DNA Elements
BWA	Burrow Wheelers alignment
GFP	Green fluorescence protein
YFP	Yellow fluorescence protein
SET	Single-ends tags
PET	Paired-ends tags
JA	Jasmonic acid
CRISPR	Clustered regularly interspaced short palindromic repeats
